# Supplementary material for: Nature-Based Interventions Targeting Elderly People’s Health and Well-Being: An Evidence Map
Source: Int J Environ Res Public Health. 2024 Jan 19;21(1):112. doi: 10.3390/ijerph21010112 (PMC10815627; doi:10.3390/ijerph21010112)
Supplement: Supplementary file 1 [file ijerph-21-00112-s001.zip › Supplementary File S1.pdf]

**Supplemental Material – Final search strategy**

**Nature-based interventions on elderly people's health and well-being: a Systematic Evidence Map**

**Giulia Catissi, Gabriela Gouveia, Roberta Maria Savieto, Cristiane Pavanello, Raquel Simões de Almeida, Gustavo Borba Benvenutti, Kaue Alves Rosario, Eliseth Ribeiro Leão.**

(((((("aged"[MeSH Terms]) AND (("nature"[MeSH Terms]) OR ("forests"[MeSH Terms]))) OR (("nature"[MeSH Terms]) OR ("forests"[MeSH Terms]) AND (aged[Filter]))) OR ((((((((((((((((((((((((((((((((((((((("nature based"[Title/Abstract]) OR ("nature therapy"[Title/Abstract])) OR ("nature contact"[Title/Abstract])) OR ("exposure to nature"[Title/Abstract])) OR ("nature interactions"[Title/Abstract])) OR ("nature relatedness"[Title/Abstract])) OR ("nature connection"[Title/Abstract])) OR ("nature connections"[Title/Abstract])) OR ("nature exposure"[Title/Abstract])) OR ("benefits of nature"[Title/Abstract])) OR ("natural environment"[Title/Abstract])) OR ("natural environments"[Title/Abstract])) OR ("connectedness to nature"[Title/Abstract])) OR ("nature effect"[Title/Abstract])) OR ("nature sounds"[Title/Abstract])) OR ("nature elements"[Title/Abstract])) OR ("access to nature"[Title/Abstract])) OR ("nature interventions"[Title/Abstract])) OR ("nature scenes"[Title/Abstract])) OR ("nature sound"[Title/Abstract])) OR ("access to nature"[Title/Abstract])) OR ("nature view"[Title/Abstract])) OR ("nature views"[Title/Abstract])) OR ("views of nature"[Title/Abstract])) OR ("nature scenery"[Title/Abstract])) OR ("activity in nature"[Title/Abstract])) OR ("connectedness to nature"[Title/Abstract])) OR ("nature experience"[Title/Abstract])) OR ("contact with nature"[Title/Abstract])) OR ("experiencing nature"[Title/Abstract])) OR ("forest therapy"[Title/Abstract])) OR ("forest bathing"[Title/Abstract])) OR ("shinrin yoku"[Title/Abstract])) OR ("green space"[Title/Abstract])) OR ("blue space"[Title/Abstract])) OR ("green environment"[Title/Abstract])) OR ("therapeutic garden"[Title/Abstract])) OR ("healing garden"[Title/Abstract])) AND ((((("elderly"[Title/Abstract]) OR ("aged"[Title/Abstract])) OR ("old aged"[Title/Abstract])) OR ("elder"[Title/Abstract])) OR ("aging"[Title/Abstract])))) OR (((((((((((((((((((((((((((((((((((((("nature based"[Title/Abstract]) OR ("nature therapy"[Title/Abstract])) OR ("nature contact"[Title/Abstract])) OR ("exposure to nature"[Title/Abstract])) OR ("nature interactions"[Title/Abstract])) OR ("nature relatedness"[Title/Abstract])) OR ("nature connection"[Title/Abstract])) OR ("nature connections"[Title/Abstract])) OR ("nature exposure"[Title/Abstract])) OR ("benefits of nature"[Title/Abstract])) OR ("natural environment"[Title/Abstract])) OR ("natural environments"[Title/Abstract])) OR ("connectedness to nature"[Title/Abstract])) OR ("nature effect"[Title/Abstract])) OR ("nature sounds"[Title/Abstract])) OR ("nature elements"[Title/Abstract])) OR ("access to nature"[Title/Abstract])) OR ("nature interventions"[Title/Abstract])) OR ("nature scenes"[Title/Abstract])) OR ("nature sound"[Title/Abstract])) OR ("access to nature"[Title/Abstract])) OR ("nature view"[Title/Abstract])) OR ("nature views"[Title/Abstract])) OR ("views of nature"[Title/Abstract])) OR ("nature scenery"[Title/Abstract])) OR

("activity in nature"[Title/Abstract])) OR ("connectedness to nature"[Title/Abstract])) OR ("nature experience"[Title/Abstract])) OR ("contact with nature"[Title/Abstract])) OR ("experiencing nature"[Title/Abstract])) OR ("forest therapy"[Title/Abstract])) OR ("forest bathing"[Title/Abstract])) OR ("shinrin yoku"[Title/Abstract])) OR ("green space"[Title/Abstract])) OR ("blue space"[Title/Abstract])) OR ("green environment"[Title/Abstract])) OR ("therapeutic garden"[Title/Abstract])) OR ("healing garden"[Title/Abstract]) AND (aged[Filter]))) OR ((((((((((((((((((((((((((((((((((((((((((("nature based"[Text Word]) OR ("nature therapy"[Text Word])) OR ("nature contact"[Text Word])) OR ("exposure to nature"[Text Word])) OR ("nature interactions"[Text Word])) OR ("nature relatedness"[Text Word])) OR ("nature connection"[Text Word])) OR ("nature connections"[Text Word])) OR ("nature exposure"[Text Word])) OR ("benefits of nature"[Text Word])) OR ("natural environment"[Text Word])) OR ("natural environments"[Text Word])) OR ("connectedness to nature"[Text Word])) OR ("nature effect"[Text Word])) OR ("nature sounds"[Text Word])) OR ("nature elements"[Text Word])) OR ("access to nature"[Text Word])) OR ("nature interventions"[Text Word])) OR ("nature scenes"[Text Word])) OR ("nature sound"[Text Word])) OR ("access to nature"[Text Word])) OR ("nature view"[Text Word])) OR ("nature views"[Text Word])) OR ("views of nature"[Text Word])) OR ("nature scenery"[Text Word])) OR ("activity in nature"[Text Word])) OR ("connectedness to nature"[Text Word])) OR ("nature experience"[Text Word])) OR ("contact with nature"[Text Word])) OR ("experiencing nature"[Text Word])) OR ("forest therapy"[Text Word])) OR ("forest bathing"[Text Word])) OR ("shinrin yoku"[Text Word])) OR ("green space"[Text Word])) OR ("blue space"[Text Word])) OR ("green environment"[Text Word])) OR ("therapeutic garden"[Text Word])) OR ("healing garden"[Text Word]) AND (aged[Filter]))) OR ((((((((((((((((((((((((((((((((((((((((((("nature based"[Text Word]) OR ("nature therapy"[Text Word])) OR ("nature contact"[Text Word])) OR ("exposure to nature"[Text Word])) OR ("nature interactions"[Text Word])) OR ("nature relatedness"[Text Word])) OR ("nature connection"[Text Word])) OR ("nature connections"[Text Word])) OR ("nature exposure"[Text Word])) OR ("benefits of nature"[Text Word])) OR ("natural environment"[Text Word])) OR ("natural environments"[Text Word])) OR ("connectedness to nature"[Text Word])) OR ("nature effect"[Text Word])) OR ("nature sounds"[Text Word])) OR ("nature elements"[Text Word])) OR ("access to nature"[Text Word])) OR ("nature interventions"[Text Word])) OR ("nature scenes"[Text Word])) OR ("nature sound"[Text Word])) OR ("access to nature"[Text Word])) OR ("nature view"[Text Word])) OR ("nature views"[Text Word])) OR ("views of nature"[Text Word])) OR ("nature scenery"[Text Word])) OR ("activity in nature"[Text Word])) OR ("connectedness to nature"[Text Word])) OR ("nature experience"[Text Word])) OR ("contact with nature"[Text Word])) OR ("experiencing nature"[Text Word])) OR ("forest therapy"[Text Word])) OR ("forest bathing"[Text Word])) OR ("shinrin yoku"[Text Word])) OR ("green space"[Text Word])) OR ("blue space"[Text Word])) OR ("green environment"[Text Word])) OR ("therapeutic garden"[Text Word])) OR ("healing garden"[Text Word])) AND ((((("elderly"[Text Word]) OR ("aged"[Text Word])) OR ("old aged"[Text Word])) OR ("elder"[Text Word])) OR ("aging"[Text Word])))
